# Moonshot Science—Risks and Benefits

**DOI:** 10.1128/mBio.01381-16

**Published:** 2016-08-30

**Authors:** Arturo Casadevall, Ferric C. Fang

**Affiliations:** a Department of Molecular Microbiology and Immunology, Johns Hopkins Bloomberg School of Public Health, Baltimore, Maryland, USA; b Departments of Laboratory Medicine and Microbiology, University of Washington School of Medicine, Seattle, Washington, USA

## Abstract

Ever since the successful Apollo 11 Moon landing in 1969, a “moonshot” has come to signify a bold effort to achieve a seemingly impossible task. The Obama administration recently called for a moonshot to cure cancer, an initiative that has elicited mixed responses from researchers who welcome additional funding but worry about raising expectations. We suggest that a successful moonshot requires a sufficient understanding of the basic science underlying a problem in question so that efforts can be focused on engineering a solution. Current gaps in our basic knowledge of cancer biology make the cancer moonshot a uniquely challenging endeavor. Nevertheless, history has shown that intensive research efforts have frequently yielded conceptual and technological breakthroughs with unanticipated benefits for society. We expect that this effort will be no different.

## EDITORIAL

**Shoot for the moon, because even if you miss, you’ll land among the stars.**

—Leslie Brown (18)

President Obama’s call for a moonshot to cure cancer has elicited mixed responses from medical researchers. Many praise it, because any increase in funding for biomedical science is welcome after more than a decade of diminishing research dollars (1), but others worry that the problem of cancer is too complex and too difficult to promise a cure (2). The critics are concerned about what can realistically be accomplished against cancer and fear that failing to deliver a cure in a timely fashion risks eroding public confidence in science.

Aside from raising doubts about curing cancer, the discussion raises the larger questions of how science works and how scientific progress can be fostered. History provides several examples of large public expenditures that produced spectacular results, including the Manhattan Project in the 1940s, the Moon landing in the 1960s, the development of AIDS therapies beginning in the 1980s, and completion of the human genome project in the 1990s. On the other hand, President Nixon’s war on cancer of the 1970s failed to deliver a cure.

Why are some large research endeavors successful, while others fail to deliver? One major difference between successful and unsuccessful scientific moonshots is the extent to which the fundamental basic science underlying the goal is understood. The Manhattan Project that delivered the atom bomb in 1945 to end World War II was based on a solid theoretical understanding of nuclear fission, which had been understood since the late 1930s (3). Similarly, the moon landings of the 1960s relied on 17th-century Newtonian physics and advances made in the German ballistic missile programs of the 1940s (4). Scientists combating the AIDS epidemic benefited from decades of research on retroviruses dating back to the early 20th century (5), so that the first cases of AIDS in 1981 were quickly followed by the discovery of HIV in 1983 (6) and the advent of antiretroviral therapy in 1987 (7). Even the first antiretroviral drug, zidovudine (AZT), was a repurposed anticancer compound that was first synthesized in 1964 (8). The human genome project of the 1990s was a triumph that relied on decades of fundamental science (9). Scientists had already established that DNA was responsible for heredity, had a double-helical structure, and could be sequenced by various techniques. Technological advances greatly accelerated their efforts to sequence the human genome.

The same rules have applied to three recent public health emergencies: the severe acute respiratory syndrome (SARS) coronavirus epidemic of 2003, the West African Ebola virus outbreak in 2012, and the current emergence of Zika virus in the Americas. In 2003, a new coronavirus rapidly spread from Asia throughout the world (10). However, the SARS epidemic was contained after only a few months as the result of a rapid response that relied on well-established principles for the epidemiological control of infectious diseases, including rapid identification of cases and the isolation and quarantine of infected individuals. The full SARS genome sequence was known within weeks of the identification of the infectious agent (11). Within months, neutralizing human monoclonal antibodies (MAbs) were made to provide a means for specific treatment and prophylaxis (12). In 2014, the world experienced the largest Ebola virus outbreak in history, killing thousands of individuals in multiple countries (13). The Ebola outbreak was also controlled through infection control protocols that reduced contagion, including the strict isolation of infected patients and the use of full personal protective equipment. Once again, new therapeutics in the form of passive transfer of MAbs and immune sera were used, and a vaccine was developed so rapidly that it could be tested in the final stages of the outbreak (14). In both the SARS and Ebola emergencies, the development of antibody therapies relied on decades of basic-science studies on antibody-mediated immunity, immunoglobulin structure, and the development of MAb technology. The world now faces the threat of Zika virus, which emerged in the Americas in 2015 to cause a constellation of diseases ranging from microcephaly to Guillain-Barré syndrome (15). Substantial resources are being considered to combat this menace, and rapid mobilization to test antiviral compounds, to obtain neutralizing antibodies for prophylaxis and therapy, and to develop vaccines is possible only due to an existing scientific infrastructure that can build on prior knowledge to help society address a new menace.

Although the challenges associated with landing on the Moon and controlling a viral epidemic are very different, each of the above examples shares a common denominator: projects can ultimately succeed when earlier generations have invested in basic-science research, often without necessarily knowing where it will lead. Is the cancer field ready for a moonshot? Perhaps. In the 4 decades since Nixon’s war on cancer, there has been tremendous progress in our understanding of cancer, including the discovery of oncogenes, cellular growth factors, and mutations associated with carcinogenesis. Although cancer remains a major killer throughout the world, the death rates for a number of major cancers, including gastric, breast, uterine, lung, prostate, and colorectal cancer, are actually declining (16), while average survival times have improved. Even if a cure is not forthcoming, the risk of shooting for the Moon is low. Earlier moonshots have produced benefits that could not possibly have been envisaged when the projects began. The Manhattan Project generated a vast amount of spin-off information that found its way into civilian nuclear power, radioisotopes for medical use, and plutonium-based batteries for exploratory spacecraft. The space program of the 1960s improved weather forecasting through satellite observation and greatly enhanced telecommunication, and it gave us the global positioning system that empowers our cell phones. Successes in HIV treatment showed that it was possible to effectively treat chronic viral infections, and today the same technology is being applied to many different viruses, some of which cause cancer. The human genome project of the 1990s led to the development of rapid sequencing technologies that have brought molecular biology into routine clinical use, including the use of sequence information to guide cancer therapy. Although Nixon’s war on cancer failed to deliver a cure in the 1970s, that effort improved our understanding of the molecular causes of cancer, which is now bearing fruit in the form of new drugs. The efforts to contain SARS provided new information about coronaviruses, and that experience is now being applied to a new coronavirus threat known as the Middle East respiratory syndrome, or MERS (17). Even moonshots that do not reach the Moon can provide tremendous benefits for society.

The Obama cancer initiative reopens the debate on the optimal approaches for investment of public funds in biomedical research. While acknowledging the complex and formidable challenges posed by cancer, we are confident that if money for the cancer moonshot is spent on good projects, those projects are likely to yield many benefits to society, even if cures for some cancers continue to be elusive. Just as money spent on cancer in earlier years allowed rapid progress against HIV, knowledge generated from this initiative is likely to benefit many fields in addition to oncology. We urge policymakers and administrators to recall that the successful moonshots of the past each involved a solid foundation of basic science that was translated into practical applications that were useful to society. A broad research effort that balances advances in basic knowledge, the development of novel technologies, and robust clinical trials will give us the best chance to succeed. Moonshot science is grand, optimistic, and well worth the risk.
